# Synthetic data as external control arms in scarce single-arm clinical trials

**DOI:** 10.1371/journal.pdig.0000581

**Published:** 2025-01-23

**Authors:** Severin Elvatun, Daan Knoors, Simon Brant, Christian Jonasson, Jan F. Nygård

**Affiliations:** 1 Cancer Registry of Norway, Norwegian Institute of Public health, Ullernchausseen 64, 0379 Oslo, Norway; 2 NordicRWE, Universitetsgata 2, 0164 Oslo, Norway; Drexel University, UNITED STATES OF AMERICA

## Abstract

An external control arm based on health registry data can serve as an alternative comparator in single-arm drug development studies that lack a benchmark for comparison to the experimental treatment. However, accessing such observational healthcare data involves a lengthy and intricate application process, delaying drug approval studies and access to novel treatments. Clinical trials typically comprise only a few hundred patients usually with high-cardinality features, which makes individual data instances more exposed to re-identification attacks. We examine whether synthetic data can serve as a proxy for the empirical control arm data by providing the same research outcomes while reducing the risk of information disclosure. We propose a reversible data generalization procedure to address these particular data characteristics that can be used in conjunction with any generator algorithm. It reduces the input data cardinality pre-synthesis and reverses it post-synthesis to regain the original data structure. Finally, we test a selection of state-of-the-art generators against a suite of utility and privacy metrics. The external control arm benchmark was generated using data from Norwegian health registries. In this retrospective study, we compare various synthetic data generation algorithms in numerical experiments, focusing on the utility of the synthetic data to support the conclusions drawn from the empirical data, and analysing the risk of sensitive information disclosure. Our results indicate that data generalization is advantageous to enhance both data utility and privacy in smaller datasets with high cardinality. Moreover, the generator algorithms demonstrate the ability to generate synthetic data of high utility without compromising the confidentiality of the empirical data. Our finding suggests that synthetic external control arms could serve as a viable alternative to observational data in drug development studies, while reducing the risk of revealing sensitive patient information.

## Author summary

In our research, we explored how synthetically generated data can serve as a comparison benchmark to experimental treatments in drug development studies. Such studies often face challenges in accessing a benchmark for clinical trials, delaying the evaluation and potentially the access to novel treatments. We examined whether synthetic data can serve as a proxy for an empirical benchmark, providing the same study evaluation results while reducing the risk of disclosing patient information. In our approach, we introduced a method to simplify the data before creating synthetic versions and then reconstruct the original structure after synthesis. We compared various synthetic data generation techniques to see if they could effectively replicate the utility of real data without compromising patient privacy. Our findings show that synthetic data can indeed match the usefulness of empirical data while reducing the risk of revealing sensitive patient information. This suggests that synthetic data could be a practical alternative in drug development. Such an advancement could speed up the process of evaluating and bringing new treatments to market while enhancing patient privacy.

## Introduction

There is a growing emphasis on leveraging observational data in drug development studies. The observational data may serve as an external control arm in lieu of a benchmark for comparison to the experimental treatment (active arm). The ideal scenario involves accessing data from a control arm collected in the same meticulously controlled environment as the active arm in a clinical trial. However, there are instances where randomizing to a control arm is impractical or ethically challenging. In such cases, observational data could be an alternative [1].

The U.S. Food and Drug Administration has released guiding documents outlining procedures for conducting clinical trials that utilize observational data instead of a control group, often referred to as an external control arm. To construct such a control arm, researchers have explored using data from the Cancer Registry of Norway (CRN) as a substitute comparator arm in clinical trials [2]. However, accessing observational medical data remains challenging while staying in alignment with the applicable privacy regulations and the risk of disclosing sensitive information.

Across various countries, especially within the EU, stringent legal requirements mandate minimal exposure of sensitive personal data. Consequently, gaining access to these data sources often involves navigating through a lengthy and intricate application process. In the context of drug approval, surmounting this procedural hurdle could potentially lead to delays in accessing novel treatments.

An alternative approach to using the observational data involves synthetically generated data. Synthetic data are artificial instances drawn from an approximate distribution of the empirical data. Given sufficient resemblance between the synthetic and the original data, the approximation can serve as a replacement for the original data to analyse treatment efficacy. Furthermore, synthetic data is devoid of one-to-one relationship between a synthetic record and an actual patient, reducing the risk of disclosing sensitive information. Thus, an external control arm constructed from synthetic data offers an alternative approach when accessing observational data is subject to strict privacy and legal constraints.

To investigate the potential for leveraging synthetic data in clinical trial studies, we use data from nation-wide healthcare registers. Our goal is to assess whether using this synthetic control arm together with data from the active arm can support the same conclusions as the empirical data, while also reducing the risk of information disclosure. We compare different methods for synthetic data generation, and evaluate the utility and privacy of the synthetic control arm to evaluate whether it could be a viable alternative to the original data.

## Methods

To create a synthetic external control arm, we draw a set of artificial instances from an approximation of the empirical data distribution. This approximation is derived by fitting a generative algorithm to the observational data. Using the fitted algorithm to sample artificial records, we create a synthetic dataset that closely resembles the empirical data distribution, but without replicating the original information. Various methodologies are available to model the distributions and structure of the source data. To identify the generator algorithms capable of capturing the specific data characteristics and requirements of our use case, we compare different types of algorithms.

### Synthetic data generation

Obtaining access to data from cancer registries requires the submission of a data request with a research proposal. While registries may record data in various formats, in general only aggregate or de-identified tabular data are released as other forms of data tend to have more unique personal-identifiable information. Hence, in line with the data request process, the data we consider in our application is in tabular format with patients as rows and features as columns, including numerical (e.g., age) and categorical (e.g., gender) data types.

#### Data generator selection

A generator aims to capture the distributions and relationships between features to create a synthetic dataset that mimics the properties of the original data. These generators may vary greatly in terms of their modeling approach, making some better suited for particular data types and requirements.

In preliminary studies, we experimented with various approaches, including Bayesian Networks, Variational Autoencoders, and Generative Adversarial Networks (GANs), and found that GANs provided the most robust utility. They also support rigorous privacy definitions through mechanisms such as differential privacy. Based on reviews investigating the state of synthetic tabular health data [3], the majority of new publications and applications seem to have shifted towards synthesis methods based on GANs. Therefore, for the sake of brevity and building on prior work, we focused our study primarily on adversarial methods, evaluating their performance on synthetic clinical trial data. Moreover, as private synthetic data is one of the key motivations for this study, we also include another approach that is optimised for this particular objective, namely PrivBayes. This algorithm received the Sigmoid 2024 Test of Time Award [4], having been used in various applications and is acknowledged as the first practical solution for privacy-preserving synthesis. We therefore limit our scope on the following generator selection.

The *Conditional Tabular Generative Adversarial Network* (CTGAN) is a machine learning model designed for generating synthetic tabular data, especially where the data includes mixed types (categorical and continuous) and complex dependencies between features. It is an extension of the Generative Adversarial Network (GAN) to accommodate the characteristics of tabular data [5].

Furthermore, *Survival CTGAN* is a specialized extension of the Conditional Tabular Generative Adversarial Network (CTGAN) designed to generate synthetic survival data, which includes time-to-event information and censoring indicators. It aims to produce realistic and statistically similar synthetic datasets for survival analysis [6].

Despite the potential of synthetic data in protecting sensitive information, solely relying on it may not fully address privacy concerns [7–9]. Complementary techniques, such as noise injection based on differential privacy, offer additional privacy protection [10]. Differential privacy frameworks integrated into data generator algorithms provide mathematical guarantees for privacy-preserving synthetic data generation.

The *Differentially Private Generative Adversarial Network* (DP-GAN) is a type of GAN designed to generate synthetic data while preserving the privacy of the individuals in the training dataset. It integrates differential privacy mechanisms to ensure that the synthetic data does not reveal sensitive information about any specific individual in the original data [11].

The *Private Aggregation of Teacher Ensembles* (PATE) GAN is a method designed to enhance the privacy and security of Generative Adversarial Networks (GANs) by integrating the PATE framework. PATE utilizes multiple teacher models trained on disjoint subsets of sensitive data. These teachers collectively guide a student model without exposing individual data points, thus preserving privacy. Overall, PATE GAN combines the generative capabilities of GANs with robust privacy protections, making it suitable for scenarios where data privacy is paramount [12].

Finally, *PrivBayes* is a privacy-preserving algorithm designed to generate synthetic data that maintains the statistical properties of the original dataset while ensuring individual privacy. It uses a Bayesian network to model the data distribution and incorporates differential privacy to protect sensitive information. It constructs a Bayesian network to capture the probabilistic relationships between attributes in the dataset [13].

These listed algorithms provides a comprehensive selection of techniques for tabular data generation with differential privacy. However, protecting sensitive information can still be challenging when the source data is limited in quantity. In such cases, the original dataset typically includes a few instances with distinct variations, carrying a higher privacy risk due to the exposure of their distinguishable characteristics. However, by combining synthetic data generation with other traditional privacy-enhancing techniques, statistical patterns that apply to larger populations can be emphasized while the patterns specific to individuals are masked.

### Reversible data generalization

Clinical drug trials typically comprise only a small number of patients, which makes individual data instances more exposed to re-identification attacks [14]. If the data in addition contains high-cardinality features, such as age and event horizon, the patient scarcity poses a challenge in accurately capturing statistical relationships between the features. Preserving the feature relationship information is important for the synthetic data utility, but here the data contains only a few examples of genuine feature combinations.

To address these challenges in synthesizing scarce and high-cardinality data, we use a method for *reversible data generalization*. Data generalization is a common approach for data de-identification, e.g., k-anonymity [15]. In the context of synthetic data generation, generalization can be used to reduce the cardinality of the features in the input data and thus enhance the signals on genuine feature relationships. However, this does alter the dataset properties, where the original variable range is lost. Hence, we propose a method to reverse the generalization in the synthetic data, so that the structure of the original data is regained.

Data generalization performs a mapping *x* ↦ [*b*_*n*_, *b*_*n*+1_) of each original feature value *x* into one of *N* different bins, ${\left\{\left[b_{n},\;b_{n+1}\right)\right\}}_{n=0}^{N-1}$. This reduces the feature to have cardinality *N*, and simplifies the information about joint feature distributions that must be captured by the data generators. The number of bins and their ranges can be determined based on domain knowledge or by observing statistical distributions in the dataset, e.g., selecting bins based on variable quantiles to ensure an equal number of samples in each bin.

Following the generalization and synthesis, we apply the reverse generalization procedure to the synthetic data, restoring variable cardinality to the original range. The reverse-generalization produces new feature values $\hat{x}∼D_{n}\left(\phi \right)$ from a parametric distribution *D*_*n*_ over [*b*_*n*_, *b*_*n*+1_). For binned continuous features, we take *D*_*n*_ to be a truncated normal distribution and *ϕ* to be the mean and standard deviation. To avoid disclosing sensitive information about the original data with this procedure, we use the Laplace Mechanism to estimate *ϕ* in a differentially private manner [16]. This mechanism adds noise to the output of a deterministic function, which is drawn from the Laplacian distribution and calibrated using the sensitivity of the function and the privacy budget. The privacy budget is relatively low, as only the mean and standard deviation need to be computed for the truncated normal distribution. However, with relaxed privacy guarantees, a more sophisticated density estimation function could be considered as well.

The data generalization method enables the generator to identify and model the stronger statistical patterns, while also reducing the privacy risk of individuals with low-frequency values. However, generalization does come at a cost, where more granular patterns are likely to be lost and the structure of the data is altered. Nevertheless, the reversal of this process using the parametric distribution of the generalized variable, ensures that the original variable range is restored and minimizes the effect of granular patterns being lost.

### Synthetic data quality assessment

A synthetic control arm must retain essential characteristics of the original dataset to serve as a viable replacement. Evaluating its resemblance to the original data requires an examination of pattern consistency and utility in specific applications. In addition, the privacy risk of releasing the synthetic data need to be investigated to determine whether no sensitive information about the original data subjects has leaked. The challenge is to strike the right balance between these conflicting requirements. To evaluate utility and privacy, we use a suite of metrics commonly applied in synthetic data evaluation, along with metrics that test for statistical properties typically analyzed in clinical trial datasets.

#### Data resemblance

Determining whether synthetic tabular data is able to adequately mimic the statistical patterns of the original data requires an examination of both the univariate and multivariate distributions. We employ the Kolmogorov-Smirnov (KS) statistical test, and the Jensen-Shannon (JS) distance to evaluate the similarity in between synthetic and original feature distributions. Additionally, we train separate classification algorithms on the original and the synthetic data and compare their accuracy to assess whether the feature relationships are preserved. More details on data resemblance measures are given in S1 Appendix, Section Data resemblance measures.

#### Utility

For synthetic data to be a useful control arm, it needs to support the same conclusions as the original observational data. We design four performance measures based on methods that are commonly used for comparing treatment effects to assess whether the same outcomes are achieved across both datasets. These measures aim to quantify the agreement between the synthetic and the original data in treatment efficacy estimation.

First, the *Cox beta distance* measures the difference in hazard ratios between original and synthetic data. Second, *median survival distance* calculates the absolute difference between median survival estimates from Kaplan-Meier estimators. Third, *survival curve distance* compares full survival curves, quantifying resemblance by integrating over the difference region between original and synthetic estimates. Finally, *predicted survival distance* evaluates the predictive abilities of the data by comparing patient-specific survival curves from flexible parametric models. For these four measures, a lower distance suggests a higher resemblance between the synthetic and original data for their specific task. See S1 Appendix, Section Utility measures for further information on the utility measures.

#### Privacy

Synthetic data may reduce privacy risks but does not guarantee protection of sensitive information.

With a scarce number of instances in the original data, statistical patterns are inherently more dependent on particular instances being part of the dataset. This is a concern as in particular minority groups are shown to be more susceptible to privacy risks [9]. To indicate the risk of re-identifying original information from synthetic data, we leverage four privacy measures.

Firstly, the *CAP Categorical score* evaluates the risk of inferring unknown feature values from synthetic data based on access to a few original categorical features, where a high score indicates safety from such inference attacks. Secondly, *XGB Detection* employs a XGB classifier to detect re-identification risk by classifying synthetic and original records, with successful classification suggesting lower re-identification risk. Thirdly, *Distance Closest Record* (DCR) measures the median distance from synthetic instances to the closest original instance, indicating a greater risk of memorization of training data if the synthetic dataset has a lower DCR compared to a independent hold-out set of original data. Finally, *Nearest Neighbor Distance Ratio* (NNDR) extends DCR by assessing the relative distances between the first and fifth closest data points, with a low score indicating proximity to sparsely-populated regions in the original dataset, posing a greater privacy risk. Additional details on the utility measures are given in S1 Appendix, Section Privacy measures.

### Ranking synthetic data generators

We compare generators by assigning rank scores based on their performance, similar to [17]. First, we rank the generators according to each performance measure, assigning discrete values indicating their relative performance. These scores are then aggregated into the average per generator. Subsequently, we normalize the scores to a unit range of [0, 1]. This makes the rank scores proportional to the relative generator performance, where lower scores signify better performance. Details on computing the rank scores is given in S1 Appendix, section Generator rank scores.

### Numerical experiments

We conduct numerical experiments in a retrospective analysis to investigate the impact of substituting observational data with synthetic data as the external control arm in a drug development study. Our objective is twofold: firstly to ascertain the consistency drawn from the study using the original dataset when employing synthetic data; secondly, to evaluate the potential risk associated with information disclosure stemming from the utilization of synthetic data.

#### Data

The dataset used in this study is comprised of 645 patients who were diagnosed with advanced (locally advanced or metastatic) non-small lung cancer in Norway between the 1st of January 2017, and 30th of September 2021. The dataset was restricted to patients initiating first-line treatment with either chemotherapy (combination of a platinum-based drug, and at least one other chemotherapy drug [i.e., the control arm], *N* = 397), or Pembrolizumab as monotherapy (i.e., the active arm, *N* = 248). The target outcome is overall survival, namely the time from treatment initiation to death. Follow-up ended on the 31st of December 2021. Further details on the data are given in S1 Appendix, Section Data.

#### Performance reference bounds

To establish benchmarks for generator performance, we introduce two synthetic data generators called *Dummy* and *Uniform*. The Dummy generator essentially replicates the original control arm data, representing the upper limit of data quality. Additionally, we devise a Uniform generator, employing uniform random sampling of marginals, to set a lower performance boundary. However, we remark that over 75% of our features are binary with balanced outcomes (see Table 1 in S1 Appendix). In this case, uniform sampling might yield data that appears realistic despite its naive generation method.

#### Data generators

We include in total six generator algorithms in numerical experiments: Dummy, CTGAN, Survival CTGAN, DP-GAN, PATEGAN, PrivBayes and Uniform. The generators are based on the implementations in the synthcity open-source Python package [18]. These generators creates a good spread of methods for privacy-preserving syntehtic tabular data. Each generator is trained and evaluated in ten random initializations. This means we create ten datasets per generator. The hyperparameters for each generator, including the epsilon values for differential privacy, are given in S1 Appendix, Section Hyperparameters.

## Results

The viability of employing a synthetic external control arm in clinical trial studies is herein examined in terms of performance measures to analyze the utility of synthetic data and the potential risk of information disclosure.

### Effect of reversible data generalization

As the data is scarce and with some variables of relatively high cardinalityour first goal is to examine the effect of the reversible data generalization method using a suite of synthetic data quality measures. Note that the generalization is only applied to the variables ‘age’ and ‘survival time’, as the others have low cardinality (see Table 1 in S1 Appendix). Multiple datasets are generated for each generator type either with the usage of generalization or without.

**Fig 1:**
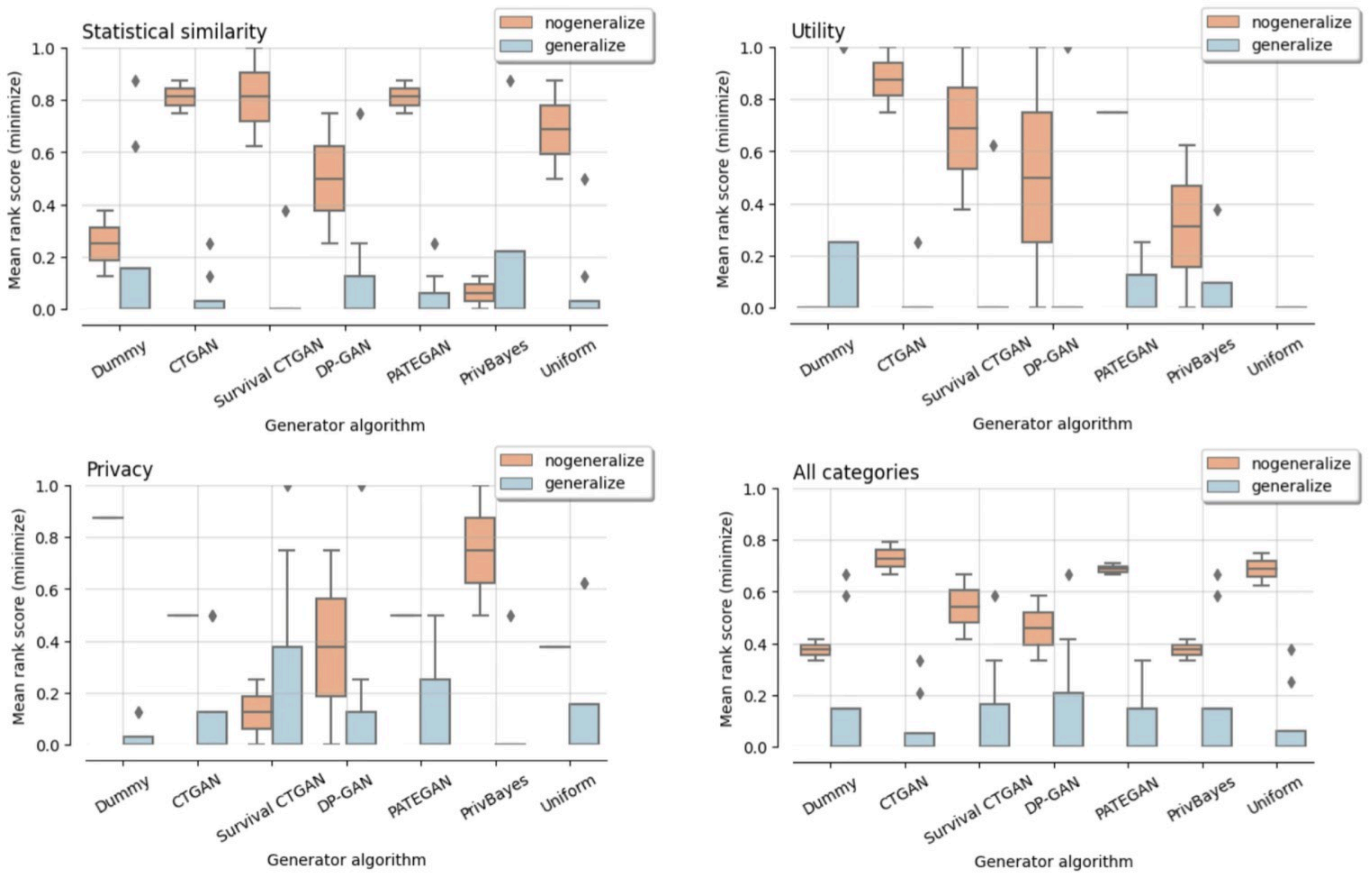
Effect of data generalization. Comparing synthetic data generated with (‘generalize’) and without data generalization (‘no generalize’).

In Fig 1, we display the rank scores of the generators for each category of data quality measures, as well as the overall ranking across all three performance measure groups. Recall that lower rank scores indicate the preferred generators.

The conclusion from Fig 1 is that the reversible generalize mechanism seems to beneficial for all generators. As expected, generalization enhances data privacy due to the aggregation of high-cardinality features prior to synthesis, and random sampling of values within the generalized domain following the synthesis. However, it also seems to improve data resemblance. Even though the granular patterns may be lost, the reduced data cardinality will likely enhance the more common signals in the data. Motivated by the improved results on data resemblance, utility and privacy, we proceed with our analyses using data generalization as pre- and post-processing.

### Synthetic data resemblance

**Fig 2:**
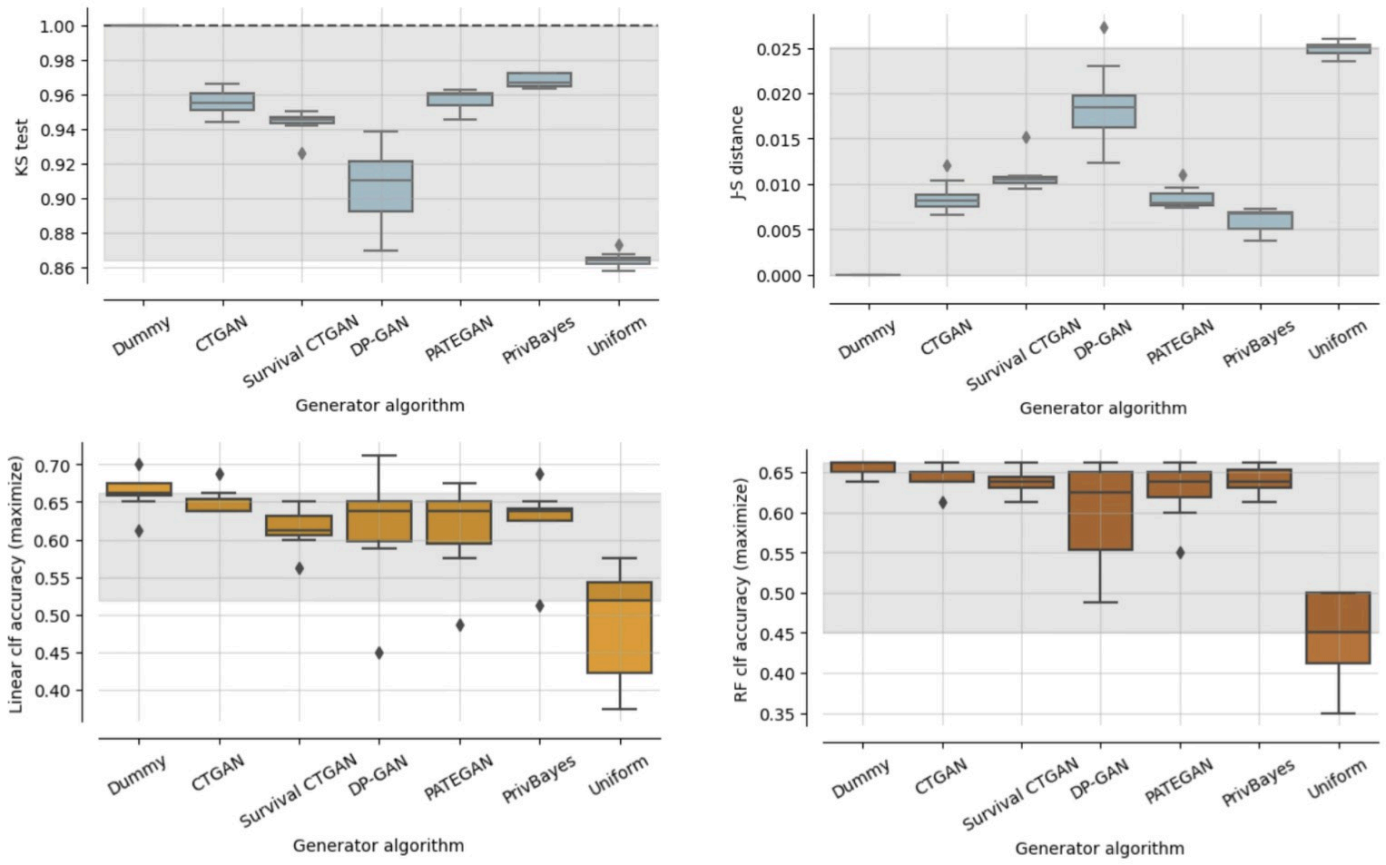
Data resemblance. Results from KS-test, J-S distance, and linear and RF classification accuracy.

As a basic check, we study the resemblance between synthetic and original data, using the data resemblance measures described in Section Data resemblance. Fig 2 shows the KS test and J-S distance scores, and the survival outcome classification scores per data generator.

Through the KS test and J-S distance, the generated data shows a strong resemblance to the Dummy generator upper baseline, with some distance from the results of the lower bound Uniform generator. Using a linear model and a random forest, their classification results are closely in line with the results on the Dummy data.

### Synthetic data utility

**Fig 3:**
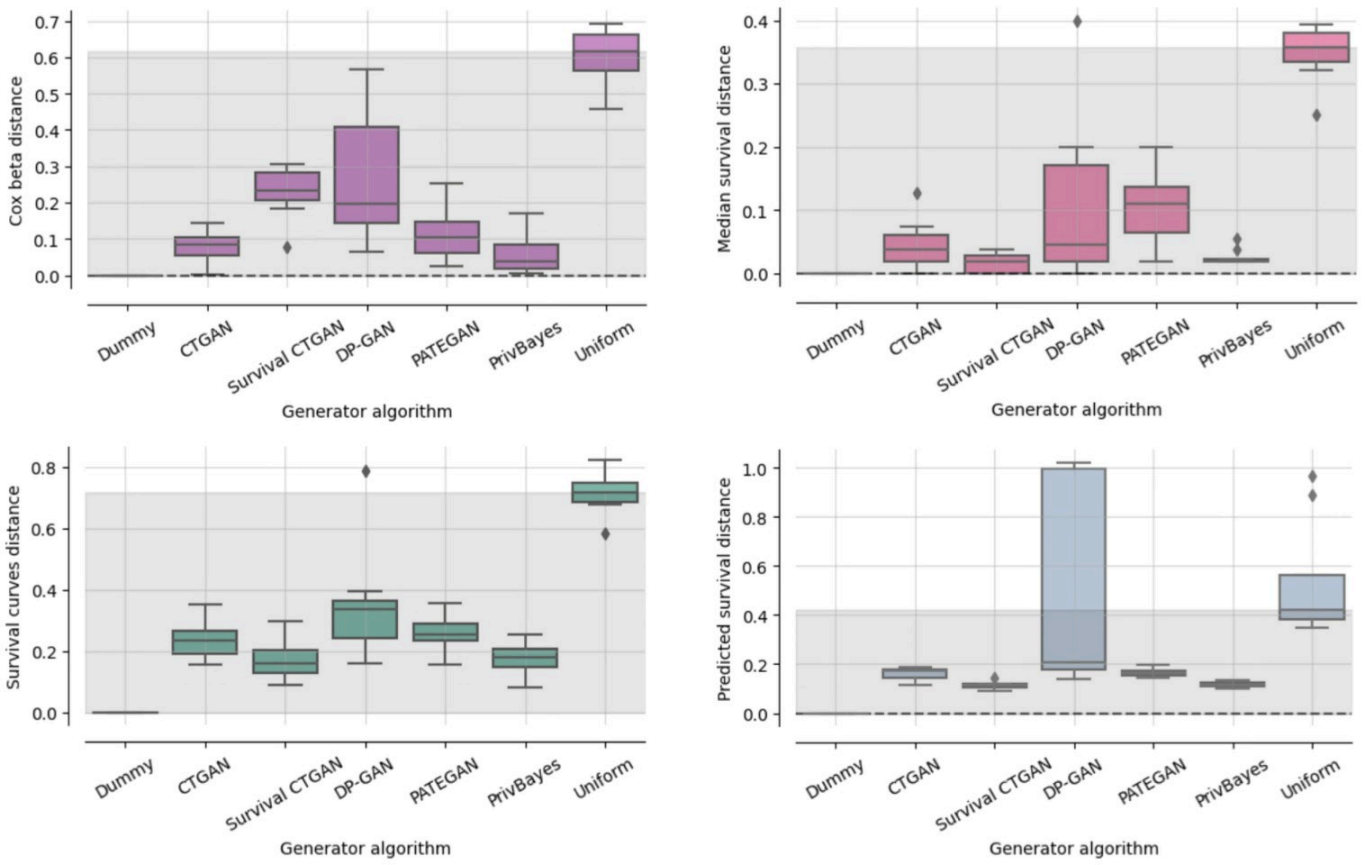
Synthetic data uility. Custom performance measures to examine the effectiveness of a synthetic external control arm.

To examine the effectiveness of substituting synthetic data with the original external control arm, we present the results for our custom performance measures in Fig 3. Note that PrivBayes, PATEGAN, and DPGAN use differential privacy, which limits privacy leakage but may also reduce data utility. (We set epsilon equal to 10 for consistency but suggest further experimentation if the respective generator is deemed satisfactory for use case.)

The Cox beta score shows the difference between hazard ratio estimates from original and synthetic data, with the difference being the smallest for PrivBayes, CTGAN and PATEGAN. Compared to the Cox beta results, the Survival CTGAN improves on the other three measures in Fig 3, comparing different aspects of survival curves. Interestingly, the PrivBayes shows competitive results with the Survival CTGAN, although PrivBayes was not designed to effectively capture information about survival outcomes.

### Privacy evaluation

The results from the previous section (Section Synthetic data utility, Fig 3) shows that most generators are capable of producing high-utility synthetic data. Generators capable of producing synthetic data that preserves the statistical information of the original data with high accuracy are expected to have greater risk of disclosing information about the original data.

**Fig 4:**
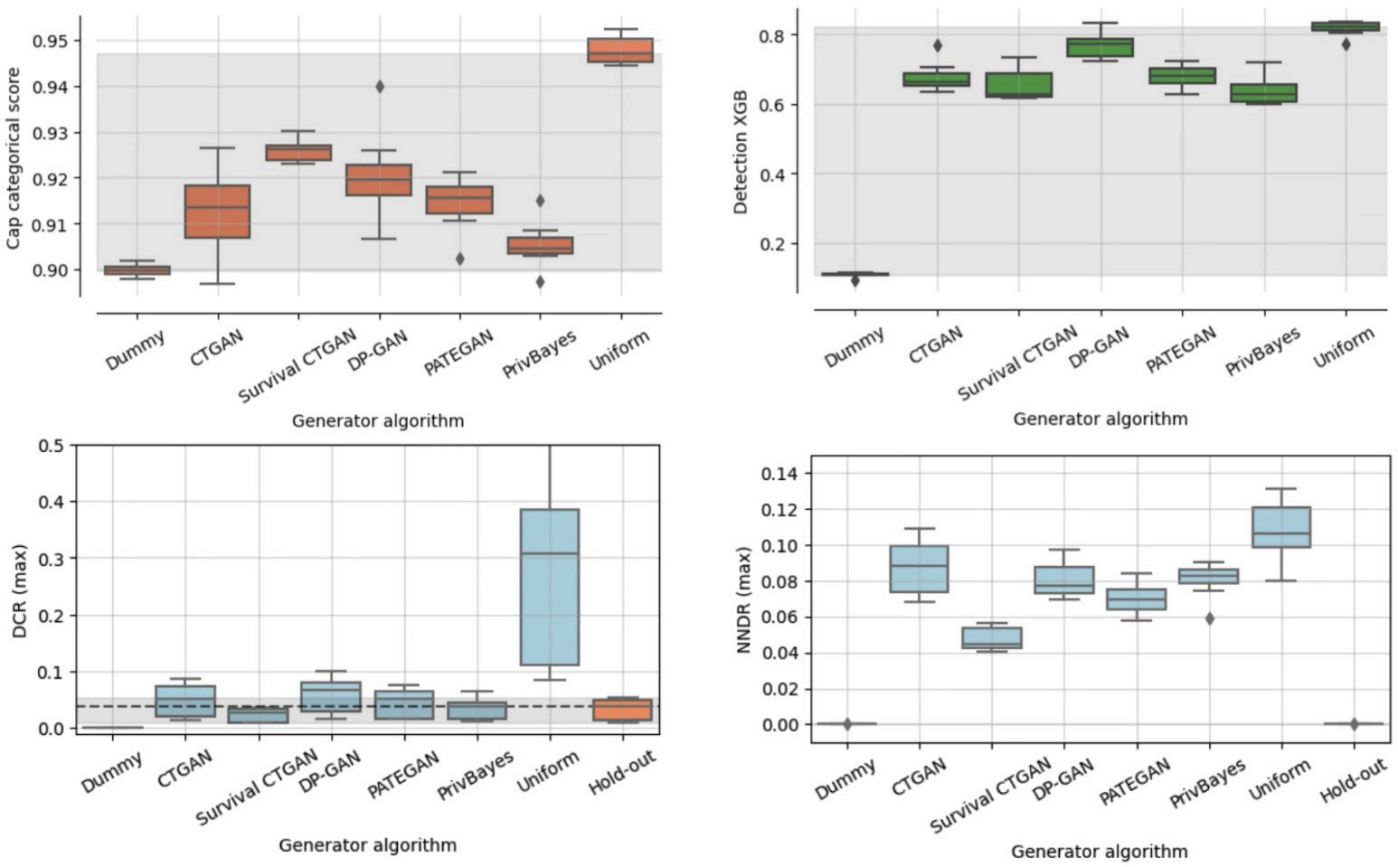
Re-identification risk. Cap Categorical, detection XGB, distance closest record (DCR) and nearest neighbor distance ratio (NNDR) scores.

To evaluate re-identification risk, we show in Fig 4 the CAP Categorical, XGB Detection, Distance Closest Record (DCR) and Nearest Neighbor Distance Ratio (NNDR) scores.

For these two measures, the Uniform generator is expected to have the lowest re-identification risk, while the Dummy generator has the highest risk.

The DCR and NNDR scores are based on the nearest neighbour distances between original and synthetic instances, and a hold-out set of instances from the original data. Here, we also include the hold-out result, showing the resemblance between the original training data and the hold-out set as baseline.

Although all generators have a high CAP Categorical score signifying low information disclosure risk, the distance to the high-risk bound (Dummy) is close terms of score range (from 0.90 to 0.95). This indicates that it is generally challenging for an attacker to infer the categorical features, even with full access to some of the other features from the original data. Also including the numerical features, the XGB shows a strong ability to distinguish between original and synthetic instances, suggesting that records in the synthetic data deviate enough from records in the original data.

Moreover, most generators have higher DCR and NNDR scores than the original hold-out data. Survival CTGAN is the only generator with a lower DCR than hold-out, indicating a higher risk of information leakage. Regarding NNDR, the hold-out appears identical to Dummy, indicating that hold-out instances tend to reside in sparse regions of the training data. This is likely due to the scarcity of patients in the original dataset.

### Generator ranking

**Fig 5:**
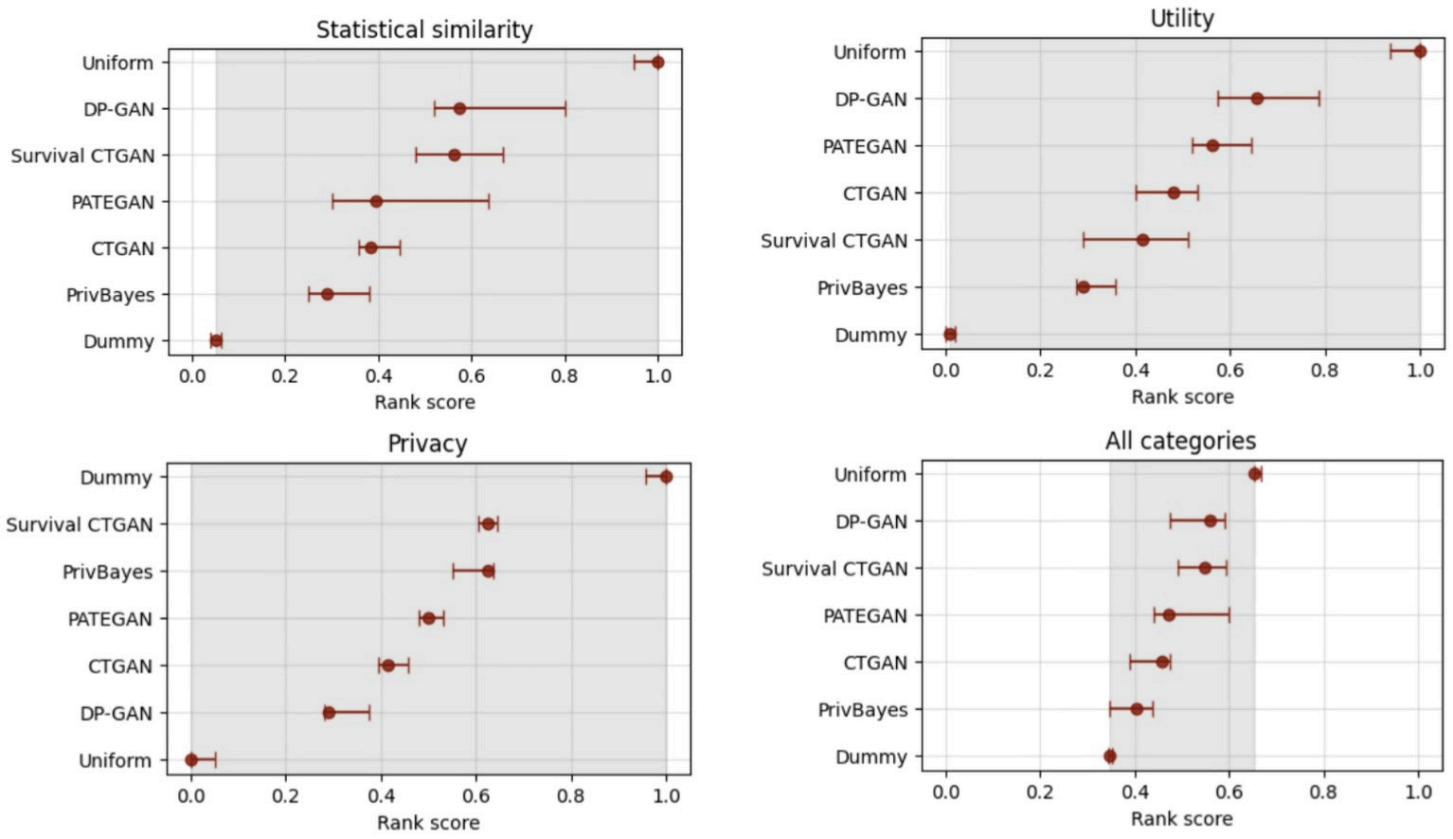
Generator selection. Ranking scores for the different metric categories and the overall ranking including all categories.

To identify the most promising generator algorithms overall, we compare the generators by their rank scores in Fig 5. The panel shows the rank scores per quality measure category, as well as the overall generator ranking across all categories. As a sanity check, we expect the Dummy generator to perform best in terms of similarity and utility (lowest rank score), while the Uniform generator should perform best on the privacy metrics.

The overall rank scores in Fig 5 shows that the PrivBayes, CTGAN and PATEGAN are the preferred generators in this study. The DPGAN generates the most private data, using the same epsilon budget as PrivBayes and PATEGAN, compromising data resemblance and utility. PrivBayes is superior in data similarity and utility but has lower privacy guarantees, while CTGAN and PATEGAN have somewhat higher privacy. However, PrivBayes has the added benefit of being able to adjust the privacy level by altering the epsilon value.

### Use-case demonstration

By constructing an illustrative example, we demonstrate the potential in leveraging synthetic control arm data to support the same conclusions obtained with the original data. In this example, we use survival curves and hazard ratio estimates to compare treatment effects. The survival curves are derived from Kaplan-Meier estimates and the hazard ratios from bootstraped Cox models. To evaluate the synthetic control arm usefulness, we visually compare the resemblance between survival curves of the chemotherapy group (control arm) and histograms of hazard-ratios. The histograms are derived from data with both the original arms and data combining the synthetic control arm with the original Pembrolizuma arm (active arm), creating a hybrid dataset.

**Fig 6:**
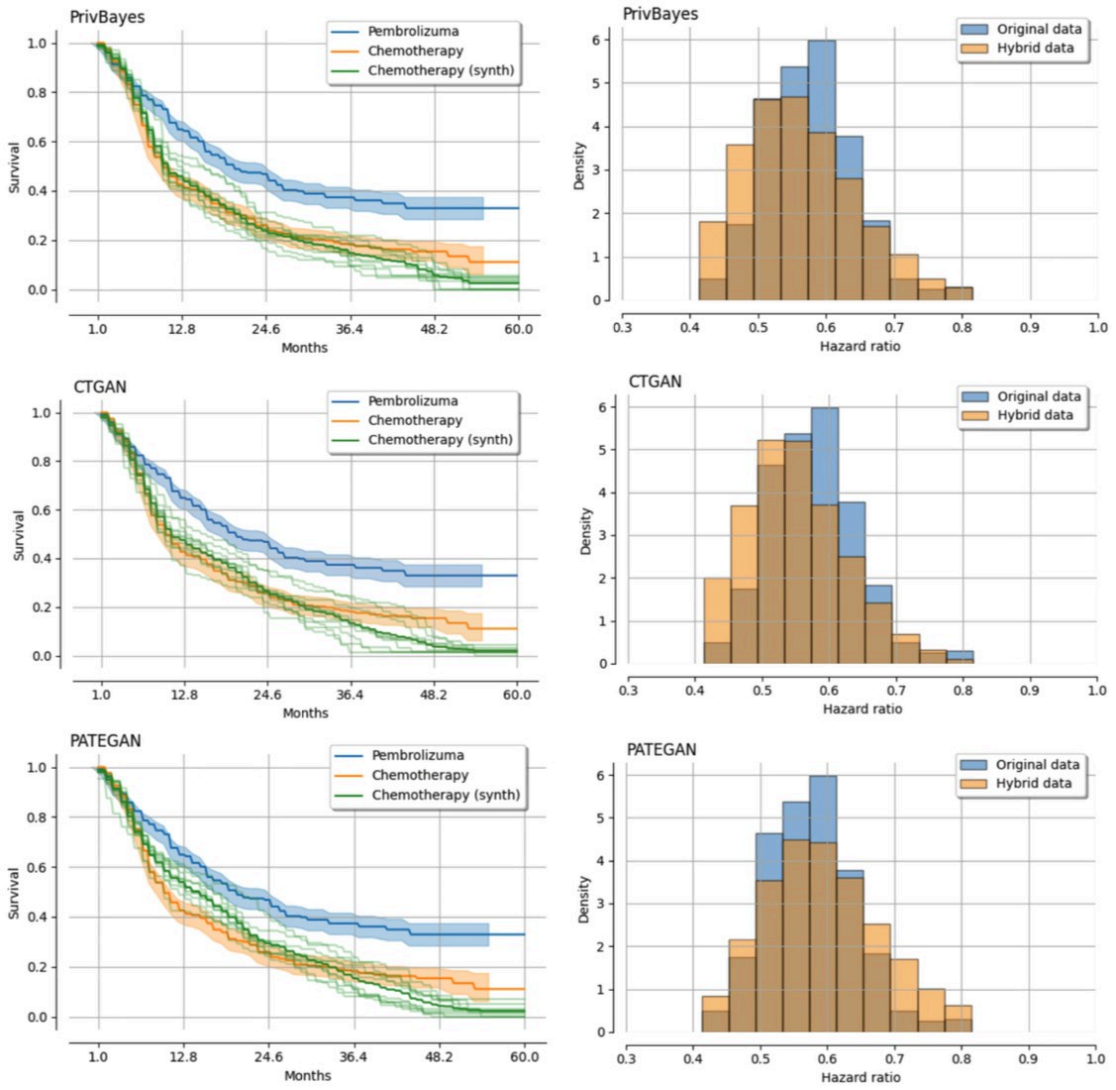
Illustrative example. Kaplan-Meier curves, and histograms of hazard-ratio estimates for the original data and hybrid data using PrivBayes, CTGAN and PATEGAN synthetic data generators.

We present the results of our illustrative example in Fig 6, using the three highest ranked generators from Fig 5. The more transparent synthetic control arm (chemotherapy) curves are from each run, while the bolder curve is the average over the runs.

We observe that the estimates from PrivBayes and CTGAN are more accurate than those from PATEGAN. Their KM curves closely align for up to approximately 25 months, after which the synthetic control arm tends to deviate slightly, showing a lower survival rate compared to the original data. Comparing histograms indicate that using data from PrivBayes and CTGAN only slightly shifts the hazard ratios towards increased survival (i.e., lower hazard-ratios than the original/reference data), while the PATEGAN data somewhat reduces survival (i.e., higher hazard-ratios). Certain discrepancies between the original and synthetic data can occur due to the inherent limitations of synthetic data generation techniques or data scarcity over certain time intervals. However, the overall conclusion regarding the difference in treatment effects remain consistent whether the synthetic or original control arm is used.

## Discussion

The aim of this paper is to indicate the potential for using synthetic data as a more accessible proxy for the external control arm in drug development studies.

Our control arm benchmark is sourced from three Norwegian health registers, and contains only a limited number of patients with some high-cardinality features. Generating synthetic data typically requires numerous instances for sufficiently strong signals on feature combinations. The reversible data generalization mechanism addressed this challenge and seemed to improve the performance for all generators. Further investigation is needed to confirm utility in other applications and datasets.

The evaluation criteria selected for this study focuses on the utility of the synthetic data to support the conclusions drawn from the original data, and the risk of information disclosure. In a comparison of different generator algorithms, PrivBayes, CTGAN and PATEGAN demonstrated the strongest capacity to produce synthetic data with resemblance to the original data with relatively low information leakage. The results suggests that synthetic control arm data can be created to mirror the conclusions drawn from the original data, while protecting sensitive information. However, despite our investigations into the risk of disclosing original data information via the synthetic data, our analyses are not sufficient evidence to conclude a safe public release of the synthetic data.

Thus, in future work, we plan to conduct a more thorough analysis of privacy requirements for granting public access to synthetic external control arms. Moreover, rather than replacing the original data, we plan to explore synthetic data augmentation for scarce data regimes in drug development studies. Based on the results from this study, we expect that augmentation will enhance the original data, leading to more decisive results.

## Supporting information

S1 Appendix Supplementary information. More detailed explanations of data resemblance measures and generator rank scores, the hyperparameters used to train the synthetic data generators, and summary statistics of the data variables.
(PDF)

## References

[1] U S Department of Health and Human Services—Food and Drug Administration. Considerations for the Use of Real-World Data and Real-World Evidence to Support Regulatory Decision-Making for Drug and Biological Products. U.S. Department of Health and Human Services—Food and Drug Administration; 2023.

[2] Børø S, Thoresen S, Boge Brant S, Helland Å. Initial investigation of using Norwegian health data for the purpose of external comparator arms-an example for non-small cell lung cancer. Acta Oncologica. 2023;62(12):1642–1648. doi: 10.1080/0284186X.2023.2264484 37801361

[3] Hernandez Mikel and Epelde Gorka and Alberdi Ane and Cilla Rodrigo and Rankin Debbie. “Synthetic data generation for tabular health records: A systematic review.” Neurocomputing 493 (2022): 28–45. doi: 10.1016/j.neucom.2022.04.053

[4] Qian, Zhaozhi, Bogdan-Constantin Cebere, and Mihaela van der Schaar. “SIGMOD 2024 Test of Time Award for ‘PrivBayes’” https://warwick.ac.uk/fac/sci/dcs/news/?newsItem=8a17841b8fe761b8019001e4d60a74e0

[5] Xu L, Skoularidou M, Cuesta-Infante A, Veeramachaneni K. Modeling tabular data using conditional gan. Advances in neural information processing systems. 2019;32. 32103879

[6] Norcliffe A, Cebere B, Imrie F, Lio P, van der Schaar M. SurvivalGAN: Generating Time-to-Event Data for Survival Analysis. In: International Conference on Artificial Intelligence and Statistics. PMLR; 2023. p. 10279–10304.

[7] Kaabachi B, Despraz J, Meurers T, Otte K, Halilovic M, Prasser F, et al. Can We Trust Synthetic Data in Medicine? A Scoping Review of Privacy and Utility Metrics. medRxiv. 2023; p. 2023–11. 10.1038/s41746-024-01359-3 PMC11772694 39870798

[8] Ganev G, De Cristofaro E. On the Inadequacy of Similarity-based Privacy Metrics: Reconstruction Attacks against” Truly Anonymous Synthetic Data”. arXiv preprint arXiv:231205114. 2023;.

[9] Stadler T, Oprisanu B, Troncoso C. Synthetic Data—Anonymisation Groundhog Day; 2022.

[10] Dwork C, Lei J. Differential privacy and robust statistics. In: Proceedings of the Forty-First Annual ACM Symposium on Theory of Computing. STOC’09. New York, NY, USA: Association for Computing Machinery; 2009. p. 371–380. Available from: https://doi-org.ezproxy.uio.no/10.1145/1536414.1536466.

[11] Xie L, Lin K, Wang S, Wang F, Zhou J. Differentially private generative adversarial network. arXiv preprint arXiv:180206739. 2018;.

[12] Jordon J, Yoon J, Van Der Schaar M. PATE-GAN: Generating synthetic data with differential privacy guarantees. In: International conference on learning representations; 2018.

[13] Zhang J, Cormode G, Procopiuc CM, Srivastava D, Xiao X. Privbayes: Private data release via bayesian networks. ACM Transactions on Database Systems (TODS). 2017;42(4):1–41. doi: 10.1145/3134428

[14] Henriksen-Bulmer J, Jeary S. Re-identification attacks—A systematic literature review. International Journal of Information Management. 2016;36(6):1184–1192. doi: 10.1016/j.ijinfomgt.2016.08.002

[15] Sweeney L. K-Anonymity: A Model for Protecting Privacy. Int J Unc Fuzz Knowl Based Syst;10(05):557–570. doi: 10.1142/S0218488502001648

[16] Dwork C, McSherry F, Nissim K, Smith A. Calibrating noise to sensitivity in private data analysis. In: Theory of Cryptography: Third Theory of Cryptography Conference, TCC 2006, New York, NY, USA, March 4-7, 2006. Proceedings 3. Springer; 2006. p. 265–284.

[17] Yan C, Yan Y, Wan Z, Zhang Z, Omberg L, Guinney J, et al. A multifaceted benchmarking of synthetic electronic health record generation models. Nature communications. 2022;13(1):7609. doi: 10.1038/s41467-022-35295-1 36494374 PMC9734113

[18] Qian, Zhaozhi, Bogdan-Constantin Cebere, and Mihaela van der Schaar. “Synthcity: facilitating innovative use cases of synthetic data in different data modalities.” arXiv preprint arXiv:2301.07573 (2023).

