# Supplementary material for: Synthetic data as external control arms in scarce single-arm clinical trials
Source: PLOS Digit Health. 2025 Jan 23;4(1):e0000581. doi: 10.1371/journal.pdig.0000581 (PMC11756779; doi:10.1371/journal.pdig.0000581)
Supplement: S1 Appendix — More detailed explanations of data resemblance measures and generator rank scores, the hyperparameters used to train the synthetic data generators, and summary statistics of the data variables. (PDF) [file pdig.0000581.s001.pdf]

S1 Appendix

Data resemblance measures

The JS distance quantifies dissimilarity between probability distributions, while the KS test evaluates disparities between a synthetic feature and the corresponding reference feature from the original data. The KS test yields a score from zero to one, where one indicates identical feature distributions, while a smaller JS distance means closer feature distributions. To calculate the KS score, we average the KS test statistic obtained from each corresponding pair of columns in the original and synthetic data, separately. Similarly, the JS distance score is calculated column-wise from corresponding columns in the synthetic and original data.

The algorithms are trained to classify the survival outcome in the control arm. A more similar classification accuracy on the the synthetic and original data indicates resemblance between the original and the synthetic data.

Utility measures

Cox beta distance Cox regression is commonly used to estimate the hazard ratio between the active and the control arm. To indicate the difference between survival outcomes inferred from the original and the synthetic data, we fit Cox models using the event horizon and treatment indicator features. A smaller absolute difference between model coefficients indicate higher resemblance between original and synthetic data.

Median survival distance Another method for treatment efficacy estimation are survival times. Here, we measure the absolute difference between median survival from Kaplan-Meier (K-M) estimators fitted to the original and the synthetic control arm. Less difference between the estimates suggests that the synthetic data captures more of the survival information.

Survival curve distance In addition to comparing median survival point estimates, we can also compare the full survival curves. Integrating over the difference region between survival curves from K-M estimators fitted to the original and the synthetic control arm quantifies the resemblance between these curves. A smaller difference region signifies that the original and synthetic estimates are more similar.

Predicted survival distance To also evaluate the generalization abilities of the data, flexible parametric models are fitted to synthetic and original control arm data, and used to predict the survival function for each patient. Here, we compare patient-specific survival curves by integrating over their absolute difference regions and averaging the results. Smaller scores signify that the original and synthetic datasets generalize equally well to survival prediction.

Privacy measures

CAP Categorical score Assuming an inference attack on the categorical features, this measure indicates the risk of inferring other features based on access to synthetic data and a few original features. To select the original features available to the attacker, we randomly sample two categorical features. If the score value equals one, the original data is considered safe from the attack.

XGB Detection Leveraging feature relationship information, we indicate re-identification risk from the classification error of an eXtreme Gradient Boosting classifier (XGB) that is trained to classify synthetic and original records.

If the XGB can distinguish between these instance classes, this suggests a lower re-identification risk. A dataset is formed by combining the synthetic and original control arm instances, which are labelled thereafter. The XGB is trained on a subset of this data and evaluated on a hold-out set in terms of the ROC AUC.

Distance Closest Record (DCR) Measures the median distance from synthetic instances to the closest original instance. The same is done for a hold-out set of original data with respect to the original training data. If a synthetic dataset has a lower DCR compared to the hold-out, then there is a greater risk that the synthetic data has memorized particular instances from the training data.

Nearest Neighbor Distance Ratio (NNDR) This measure is similar to DCR, but the distances are divided by the distance to the fifth nearest neighbor. A low score on this relative distance metric indicates that data points are close to sparsely-populated data regions in the original dataset (i.e., potential outliers) which pose a greater privacy risk.

Generator rank scores

For $1 \leq m \leq M$ measures and $1 \leq g \leq G$ generators, we organize the results from numerical experiments as a score matrix $S \in \mathbb{R}^{G \times M}$. Each entry $S_{g,m}$ is the performance score of generator g for measure m in the score matrix. To compute the rank scores per generator, the first step is to rank all G generators according to the direction of optimization (minimize/maximize) for each measure m. This ranking yields a rank matrix $R \in \mathbb{Z}^{G \times D}$, where each entry $1 \leq R_{g,d} \leq G$ is a positive integer representing the rank of generator g relative to the G other generators for measure d. To determine the overall generator rankings, we average R across the performance measures indicated on the matrix columns, producing a vector $r \in \mathbb{R}^G$ of the average rank scores per generator. The range of the rank scores depends on the number of generators G. To have bounded rank scores, we compute $\hat{r} = (r-1)/(G-1)$, producing rank scores in $[0, 1]$.

Hyperparameters

Our synthetic data generators are based on the implementations in the synthcity open-source Python package. We use synthcity version 0.2.4. Apart from the following specified parameters, we use the default parameters in the given version of the package.

The CTGAN, CTGAN Survival and DP-GAN algorithms were configured with learning rate 0.000046, while PATEGAN has learning rate 10^{-5} and delta 10^{-5}. Differential privacy generators were set with epsilon equal to 10. All neural network based generators were ran for maximum 2000 epochs.

Data

The external control arm data originates from three health registers: the Cancer Registry of Norway, the Norwegian Patient Registry and the Norwegian Prescribed Drug Registry. Table 1 gives a summary of all the variables that were included in the dataset that was used for the numerical experiments.

Patients were excluded from the dataset if (1) they were either ALK or EGFR positive; (2) they had been given any diagnoses that indicated poor organ function (such as Neutropenia or moderate to severe chronic kidney disease) in the past 2 years prior to initiating treatment; (3) they had any other primary malignancies within the past 5 years of initiating treatment.

Table 1: Summary of dataset variables.

Variable	Data type	Description	Value range	Mean	Mode
OS	Integer	Overall survival in months	1–55	14.8	3
OS status	Binary	Overall survival status	0/1	0.59	1
Treatment	Binary	Type of treatment	Chemotherapy/ Pembrolizuamb	0.38	0
Sex	Binary	Sex at birth	Male/female	0.51	0
Age	Integer	Age at diagnosis	42–90	68.9	71
ECOG	Binary	Performance status	0/1	0.61	1
Squamous	Binary	Squamous cell cancer	0/1	0.29	0
Treatment year	Integer	Year of treatment	2017–2021	2018	2018
